# Spontaneous Involution of a Thyroid Nodule: When Nodules Can’t Be Trusted

**DOI:** 10.7759/cureus.80992

**Published:** 2025-03-22

**Authors:** Sigfrido Miracle López, Manuel A Reyes Muñoz, Emilio Fernández Fernández, Luis A Lara Córdoba

**Affiliations:** 1 Center for Research in Health Sciences, Hospital Ángeles Lomas, Huixquilucan, MEX; 2 Center for Research in Health Sciences, Universidad Anáhuac México, Mexico City, MEX

**Keywords:** benign and malignant thyroid nodule, thyroid cancer, thyroid nodule, thyroid nodule size, thyroid pathology, ultrasonography, ultrasound

## Abstract

A thyroid nodule (TN) is an abnormal growth within the thyroid gland, with a large prevalence in adults. They can be classified as benign or malignant based on their characteristics. Most nodules are non-palpable and require ultrasound and histopathological analysis for identification. This study reports a rare case of TN involution despite initially exhibiting malignant ultrasound characteristics. The patient presented with a cystic TN of 27.43 x 13.31 x 35.84 mm, initially classified as Thyroid Imaging Reporting and Data System (TIRADS)-2 and Bethesda II, which later transformed into a solid mass classified as TIRADS-5 and Bethesda I. Over time, the nodule demonstrated an 88% reduction in size to 5.93 x 6.34 x 10.17 mm, while maintaining a TIRADS-5 classification. Surveillance remains the primary management approach, with a follow-up scheduled for 2025. No lymphadenopathy was detected. The observed regression supports evidence that fine needle aspiration biopsy of cystic thyroid nodules may induce necrotic and fibrotic changes, potentially due to post-procedural hemorrhage leading to hematoma formation, tissue compression, and eventual involution. The primary focus of this case review is to contribute to the relatively limited research on TN involution and to briefly discuss the underlying mechanisms.

## Introduction

Thyroid nodules (TN) have been gaining attention over the last few years because of their growing prevalence. Having an increasing number of accurate diagnostic tools, their presence can be detected by ultrasonography (USG), magnetic resonance, and axial computed tomography. Approximately 60% of the global adult population has one or more nodules, depending on the method of detection [1]. Prevalence ranges from 34% to 67%, depending on the diagnostic method used. Furthermore, risk factors such as being female, a higher body mass index (BMI), and higher age have been associated with their presence [2]. Once a TN is detected, the next step must be to rule out malignancy. Approximately 7% to 15% of nodules are found to be cancerous; the patients present with risk factors such as their sex, radiation exposure, and family history, among others [3]. The majority of TNs are not palpable; therefore, the use of USG is key for their detection, staging, and histopathology analysis, if required [4]. The use of USG is one of the most used imaging techniques for the diagnosis of papillary thyroid cancer (PTC). As a consequence, the detection of PTC globally has increased the survival rates up to 97% [2].

## Case presentation

Clinical history

A male patient, 34 years of age, presented for the first time to an outpatient medical consultation in January 2021. The patient complained of difficulty losing weight, cold intolerance, and unexplained hair loss. When questioned about his family history, he denied any thyroid disease. Past medical history included daily exercise for 60 minutes, a balanced diet, no alcohol or drug consumption, and having stopped smoking since 2016. The patient denied allergies, had keratoconus, had a fifth molar surgical extraction, and recently had a gout attack. His current medications consisted of colchicine 0.5 mg once a day (QD) and meloxicam 15 mg as needed (PRN), and the following supplements: collagen, glutamine, vitamin B complex, vitamin D3, selenium, zinc, creatine, and protein powder. On physical examination, the patient has a heart rate of 85 beats per minute (bpm), blood pressure of 110/60 mmHg, temperature of 36.6°C, oxygen saturation of 94%, height of 1.78 m, and weight of 84 kg, with a calculated BMI of 26.5 kg/m^2^. The patient's blood work showed thyroglobulin antibodies (TG-Ab) of 1.6 UI/mL, thyroid peroxidase antibodies (TPO-Ab) of 1.0 UI/mL, triiodothyronine (T3) of 105 ng/dL, thyroxine (T4) of 6.3 ng/dL, thyroid-stimulating hormone (TSH) of 2.73 UI/mL, free T3 (T3L) of 2.88 pg/mL, and free T4 (T4L) of 1.09 pg/mL. On physical examination, a solid, fixed, palpable mass was detected in the left supraclavicular region, so an in-office thyroid ultrasound (TUSG) was performed.

Therapeutic intervention 

**Figure 1:**
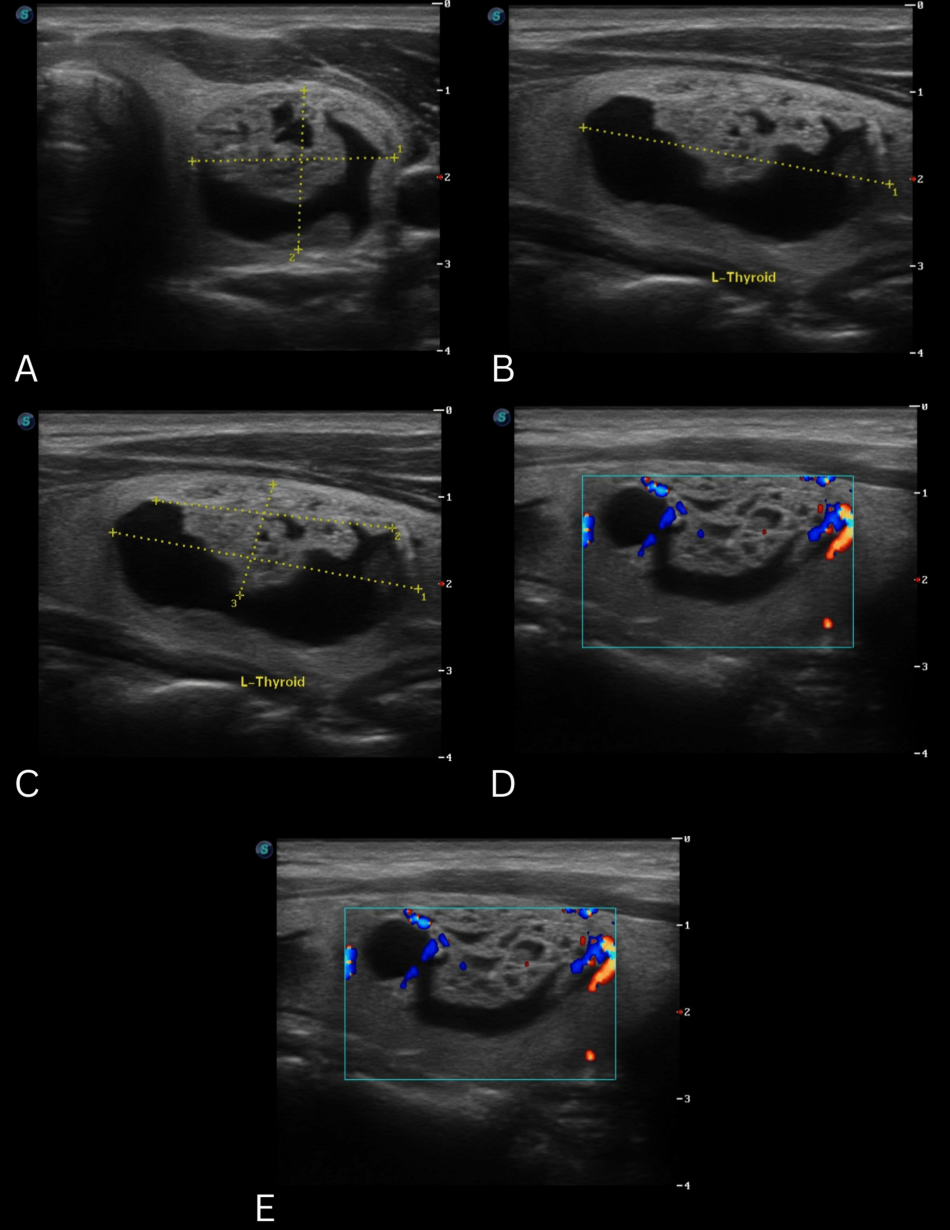
Clinical images series of TUSG 1 A. Nodular lesion in the left thyroid lobe, mixed echogenicity with a cystic predominance (1), the margins of the lesion are regular, and the margins of the solid component are non-spiculated (0); B. Wider-than-tall (0); C. Solid component isoechoic (1), with no hyperechogenic foci (0); D. Peripheral vasculature with color Doppler, short axis; E. Peripheral vasculature with color Doppler, long axis; TIRADS-2 (2 points) 27.43 x 13.31 x 35.84 mm TSUG: thyroid ultrasonography; TIRADS: Thyroid Imaging Reporting and Data System

Thyroid ultrasonography was elected as the imaging technique using the Thyroid Imaging Reporting and Data System (TIRADS) classification system [5]. Along with fine needle aspiration biopsy (FNAB) according to the TIRADS score, using the categories of the Bethesda System for Reporting Thyroid Cytopathology [5]. During the first appointment, the first TUSG reported a single mixed-solid nodule on the left thyroid lobe, classified as TIRADS-2 (Figure 1) [5]. The rest of the structures were reported without anomalies. An FNAB of the solid component and drainage of the cystic component were performed.The histopathological report of the FNAB determined the presence of a benign follicular nodule, Bethesda II [5]. No incidents were reported during the procedure. With these results, the patient remained under observation.

**Figure 2:**
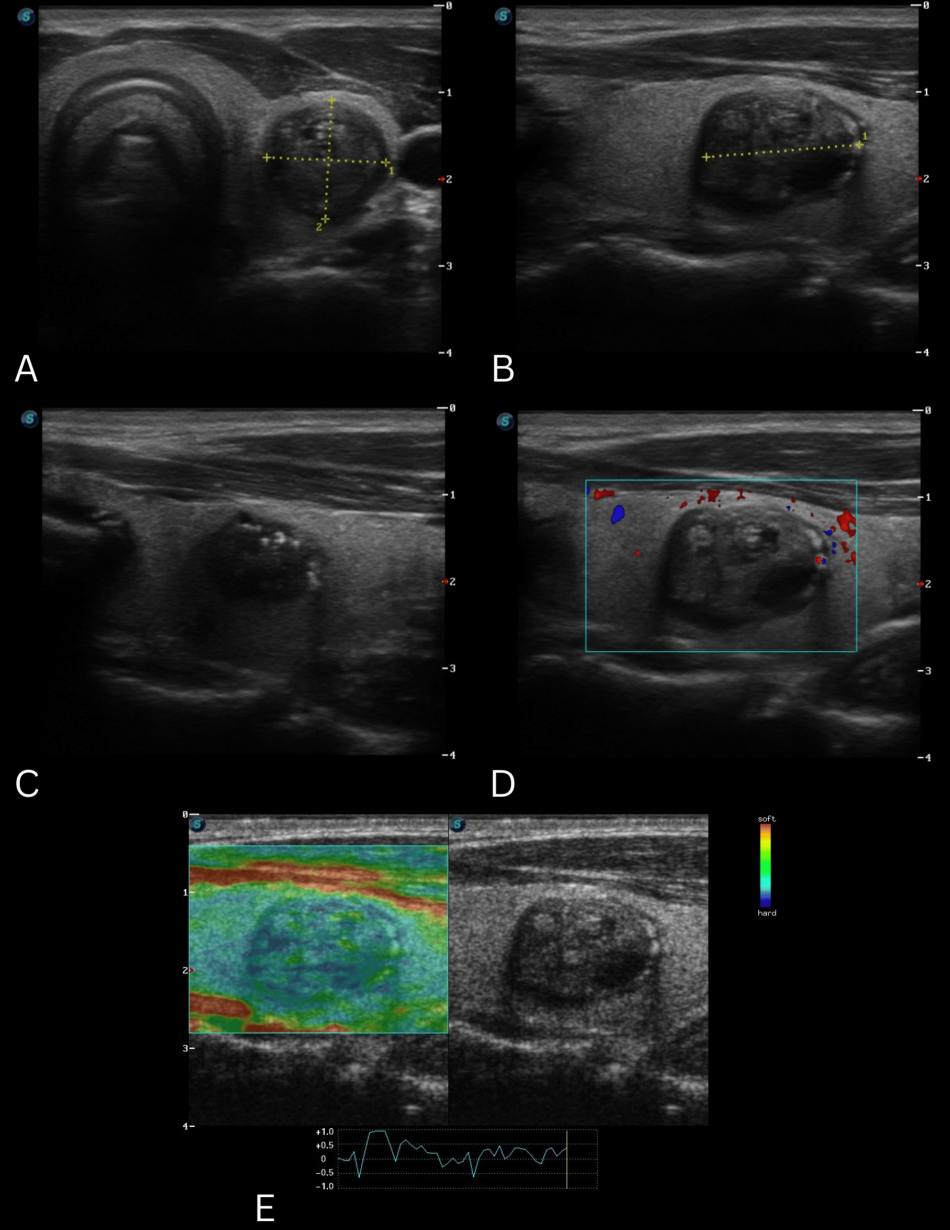
Clinical images series of TUSG 2 A. Nodular lesion in the left thyroid lobe, solid (2), hypoechoic (2), regular margins (0); B. Wider-than-tall (0); C. Incomplete eggshell calcification with irregular acoustic shadowing at the margins (2); D. Absence of peripheral vascularity on color Doppler, short axis; E. Increased stiffness on elastography under compression; TIRADS-4 (6 points) 13.76 x 13.19 x 17.72 mm TSUG: thyroid ultrasonography; TIRADS: Thyroid Imaging Reporting and Data System

The patient returned after 15 months in September 2022 for a follow-up appointment. The second TUSG reported a single nodule on the left thyroid lobe, classified as TIRADS-5 (Figure 2) [5]. The rest of the structures were reported without anomalies. It was advised to perform an additional FNAB. The pathological report described the presence of a few follicular cells with reactive changes classifying it as Bethesda I [5]. No incidents were reported during the procedure.

**Figure 3:**
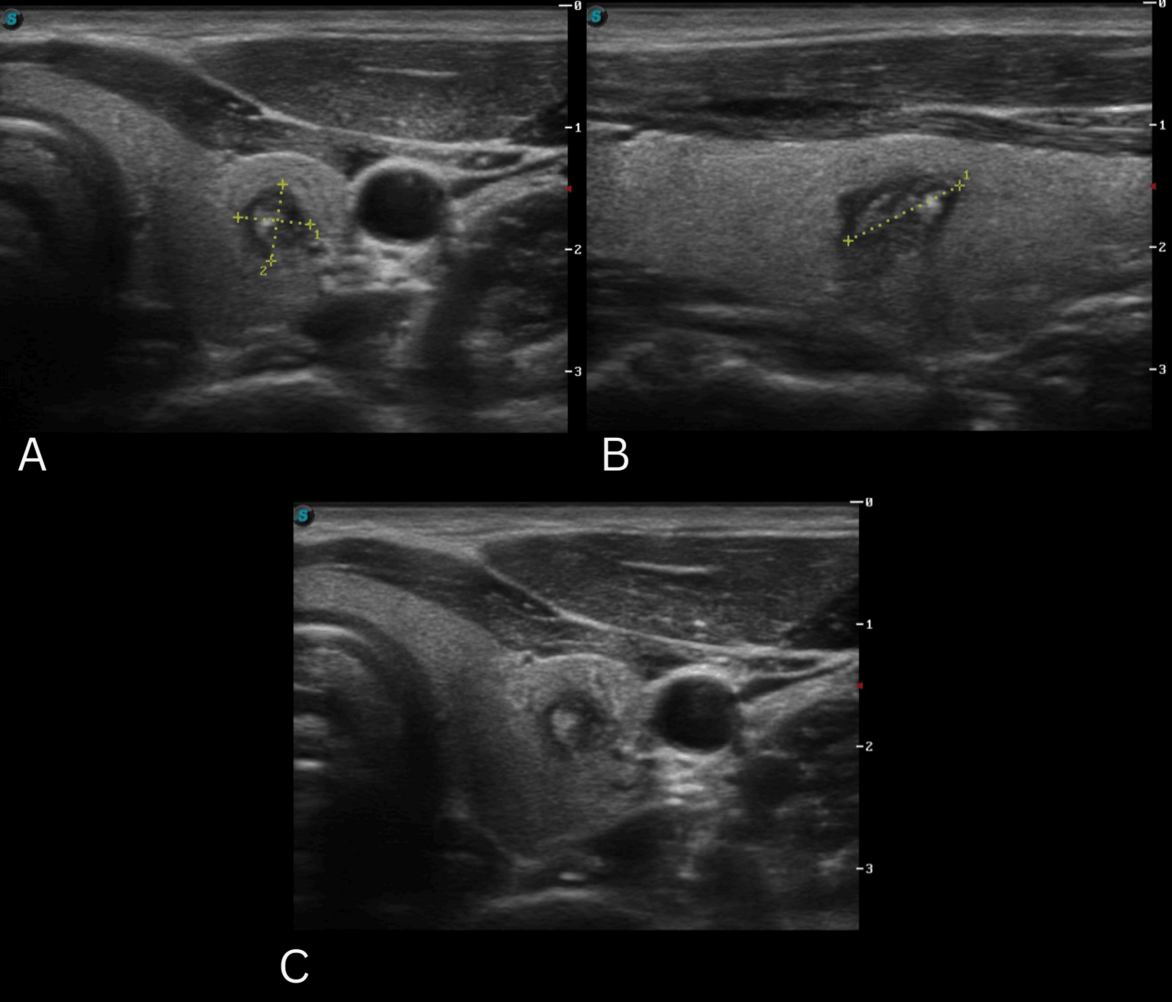
Clinical images series of TUSG 3 A. Nodular lesion in the left thyroid lobe, solid (2), hypoechoic (2), well-defined irregular margins (2)' B. Wider-than-tall (0); C. Diffuse macrocalcification (1)' TIRADS-5 (7 points) 5.93 x 6.34 x 10.17 mm TSUG: thyroid ultrasonography; TIRADS: Thyroid Imaging Reporting and Data System

The patient remained under observation and returned in 2024. The third TUSG reported a single nodule on the left thyroid lobe, classified as TIRADS-5 (Figure 3) [5]. The rest of the structures were reported without anomalies. Compared to the last TUSG, the volume of the nodule decreased by 88%. The patient was advised to come for a follow-up appointment in 2025 to perform a control TUSG.

## Discussion

**Figure 4:**
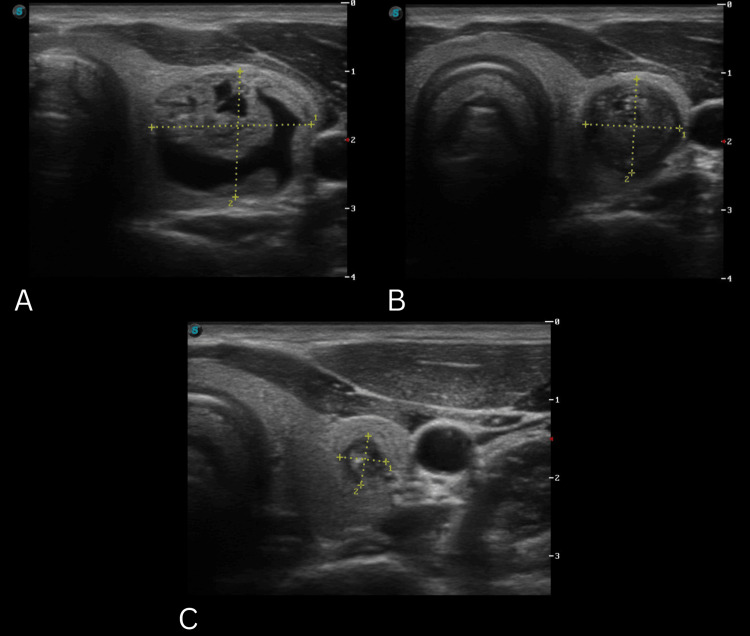
Invoution process of the thyroid nodule through appointments A. TUSG of the first appointment, in January 2021 shows a nodular lesion of 27.43 x 13.31 x 35.84 mm (6.54 cc); B. TUSG of the second appointment in September 2022 shows a nodular lesion of 13.76 x 13.19 x 17.72 mm (1.61 cc); C. TUSG of the third appointment in January 2024 shows a nodular lesion of 5.93 x 6.34 x 10.17 mm (0.19 cc); Volume reduction of 88.11% from the second to the third TUSG; Volume reduction of 97.08% from the first to the third TUSG. TUSG: thyroid ultrasonography

As can be seen in the clinical images, the TN had cystic characteristics. Initially, it was classified by TUSG as TIRADS-2 [5] and pathologically as Bethesda II [5] by FNAB. On the following evaluation, the nodule devolved into a solid mass, classified as TIRADS-5 [5] and Bethesda I [5]. Taking into account said results, the patient was seen a few months later for a control TUSG that revealed a solid mass classified as TIRADS-5 [5]. It had decreased its volume by 88%, compared to the last TUSG (Figure 4). It was decided to actively observe the nodule, and a follow-up was programmed for 2025. It's worth mentioning that lymph nodes of the laterocervical lymph chains were never found on physical examination or TUSG. The involution of the TN previously described can be attributed to various factors, and its understanding implies considering the clinical findings, imaging findings, and biopsy results. Analyzing the already existing proposed theories on the involution mechanisms of TN is essential for us to understand the ultrasonographic changes observed.

Since the 1980s, there have been clinical case reports of TN associated with the FNAB. Layfield and Loans [6] reported that following a second FNAB in two patients with different types of thyroid cancer, their results only showed necrotic and fibrous tissue. Furthermore, the mummification of TN is associated with predominant cystic characteristics, where the cyst collapses after being drained by the FNAB. Also, a possible initial involution mechanism could be the small spontaneous FNAB or biopsy-induced hemorrhage in up to 26.5% to 93.3% of the patients. The hemorrhage, combined with the formation of a hematoma, compresses the nodular tissue, and the formation of venous thrombosis induces necrosis [7]. The resulting tissue architecture change can lead to falsely elevated TIRADS as well as false malignant suspicion. To be able to rule it out, the physician must take into account the evolution of the TN in different checkups and new FNABs if necessary [8]. Ko et al. [9], in a retrospective study, reported that 46% of 13 being restructured TN had malignant characteristics in USG, like hypoechogenicity, microcalcifications, and spiked margins. Whereas Eze et al. [10], in a case series, revealed that the FNAB-induced hemorrhage in a bigger TN caused complete involution, in contrast to smaller TN that barely reduced their size. 

Considering the mechanisms of TN involution, it is important to assess the potential histopathological changes of these nodules during the post-FNAB period. Pandit and Phulpagar describe that there are concerning histological alterations after fine needle aspiration of the thyroid, or worrisome histologic alterations following fine needle aspiration of the thyroid (WHAFFT), which should be reviewed during follow-up of these nodules. It is reported that the most common acute WHAFFT findings by prevalence are hemorrhage (26.47%), capsular granulation tissue (15.68%), and the presence of siderophages (14.70%), all related to the direct trauma caused by the FNAB needle to nodular tissue at the time of puncture, leading to nodular colloid destruction and follicle obliteration. On the other hand, chronic WHAFFT findings with higher prevalence include linear fibrosis (43.13%) and nuclear atypia (11.76%). To differentiate from the appearance of ground glass in PTC, some key points in the differential diagnosis include WHAFFT lesions lacking nuclear grooves, overlapping nuclei, and showing focal presence only adjacent to the FNAB needle tract [11].

One of the most important considerations during the observation of these involuting nodules is to rule out malignancy. Using USG, several criteria have been described to differentiate benign nodules from potential PTC, primarily based on morphological parameters. These parameters include assessing the shape of the nodule, whether it is oval, round, or wider than tall, as well as evaluating the margins for spiculated outlines, poorly defined borders, hypoechoic halos, or internal isoechoic rings. Among these parameters, hypoechoic halos and hypoechoic internal rings have been described as the most sensitive, with promising negative predictive values. Additionally, the presence of posterior acoustic shadowing could suggest benignity, indicating an interface created by involution through encapsulation or peripheral fibrosis. However, the literature indicates that relying on a single diagnostic technique is insufficient to exclude malignancy, advocating for additional diagnostic tools such as biopsies and cytological studies [7].

Due to the difficulty in differentiating between involuting benign nodules and malignant nodules, dual use of USG and FNA biopsy is recommended. Fine needle aspiration biopsy alone tends to be inadequate in diagnosing these degenerative nodules due to cellular scarcity and abundant peripheral fibrosis. It has been noted that when there is discordance between these diagnostic methods, with a positive biopsy result for malignancy and suspicious findings on USG, the risk of malignancy increases between 13.6% and 56.6%. Therefore, the use of other cytological study techniques, such as core needle biopsy (CNB), is recommended, which has a positive predictive value as it can obtain more nodular tissue in a single sampling. It has been reported that, unlike discordant results between USG and FNA biopsy, discordance between USG and CNB results in a malignancy risk of only 3.6% to 5.5%, which is notably lower [7].

## Conclusions

The detailed follow-up and individualized approach in the management of TN can enable the identification of nodule-specific involution processes, offering an opportunity to adjust therapeutic interventions and avoid invasive procedures. This case contributes to the existing literature by providing a valuable perspective on the potential mechanisms of involution in TN and highlighting the importance of an adaptive management strategy based on the clinical and ultrasonographic evolution of the nodule. Further research into the mechanisms of TN involution is needed, as it may enhance current diagnostic frameworks and contribute to a more nuanced approach in TN management and the interpretation of a TUSG with malignant characteristics. In conclusion, the dynamic evolution of TN underscores the necessity of continuous monitoring and personalized management strategies, especially after FNAB due to the ultrasonographic changes it may present. 
